# Dr. Otto's Compendium of Human and Comparative Pathological Anatomy

**Published:** 1832-10-01

**Authors:** 


A Compendium of Human and Comparative Pathological Anatomy. By *Adolph Wilhelm Otto*, M.D. Royal Medical Counsellor in the Medical College of Silesia, &c. &c. Translated from the German, with additional Notes and References, by *John F. South*, Lecturer on Anatomy at St. Thomas's Hospital. Octavo, pp. 456. London, 1831.

This excellent work has only been lately placed in our hands, and we cannot delay expressing the high opinion we entertain of it. It is a refreshing thing to turn from the contemplation of works like that reviewed in our last article to productions of sterling merit. A compendium like this is of course insusceptible of analysis or review, and can only admit of a notice in a periodical journal. But it is not on that account the less valuable, and we do not hesitate to say that no scientific physician or practical surgeon should want it in his library.

In order to afford an idea of the nature of the volume, we first shall present the subjects treated as they appear upon the page of contents, and then subjoin a specimen or two of the mode in which those subjects are handled. We have little to say in addition, but we cannot refrain from expressing our satisfaction at the manner in which the original of Dr. Otto has been translated by Mr. South. The latter gentleman is already favourably known to anatomical students, and the present publication will enhance his claims to their approbation. The Borough and London University Schools are at present taking a decided lead in the medical education of the metropolis, and when we turn our eyes to the West, we look in vain for even the shadow of the glory of Baillie and the Hunters. New buildings indeed we see, but the whitewash upon them is fresh, and the memory of former days finds no sacred spot to linger in. It is with feelings of melancholy that we see the West End School, to which our own youthful recollections are attached, vailing its head to the increased intelligence, and activity, and talent of the East.

Vale, vale domus nostra !

But we turn to another, and, may we be permitted to say, a less painful subject. General anatomy has been hitherto neglected in this Country ;—there is no denying nor apologizing for the fact. Our students were *ground* for the College; and, after shuffling through the examination of that body, they hurried into practice, regardless of all but the means of making money. Hence the grovelling, ignorant, abject, contemptible creature, which the world often found the routine practitioner, and hence the debasing but far too faithful picture which the novellist and dramatist have drawn. With the great and general improvement of medical education, the method of teaching anatomy may readily be supposed to have undergone some alterations. Those alterations have been partly for the better, partly for the worse. Special anatomy is taught less rigorously, but general anatomy is more regarded. Till very lately we had no English system of general anatomy, but lately Grainger's has been published, and this was followed by the pathological anatomy of Craigie. We should not be doing justice to them if we did not express our approval of their labours—we should not be doing justice to ourselves, if we said that they are what the honour of English medicine demands. They are *not* national works, they are not to us what Bichât's was to France. Under existing circumstances we must be content with what we can get, and original productions being scarce, we must e'en import from abroad, for a good translation is not the less useful because it humbles our national pride, and exposes our national weakness. The causes of the decay of anatomical research amongst us are neither very numerous nor obscure. They are to be found in the present constitution of society in this kingdom, in the circumstance of wealth being considered the great good, whilst only successful practice can lead to it. Hence ambitious and talented men leave the dissecting-room for appointments to hospitals, and the career of private practice. This is a subject deserving more attention than it has received, but we cannot pursue it any farther at present.

The work before us is divided into two parts, one general, the other devoted to the consideration of the particular organs and tissues. The contents of the first part are as follows:—Of the Vices of Animal Organization in general—Of Vices relating to Number—Of Vices relating to Size—Of Vices relating to Form—Of Vices relating to Position—Of Vices relating to Connexion—Of Vices relating to Colour—Of Vices relating to Consistence—Of Vices relating to Continuity—Of Vices relating to Texture—Of Vices relating to Contents. From the foregoing enumeration it will be seen how comprehensively the general question of the vices of organization is considered. We shall now shew the contents of the Second, or Particular Part.

### FIRST BOOK.

#### OF THE PARTICULAR ORGANS, ON THE ORGANIC SYSTEMS.

" TWELFTH SECTION. Of Cellular or Mucous Tissue.

THIRTEENTH SECTION. Of Cellular Membranes.—*First Chapter.* Of Serous Membranes. *Second Chapter.*—Of Mucous Membranes. *Third Chapter.*—Of External Skin.

FOURTEENTH SECTION.—Of Horny Tissue. *First Chapter.*—Of the External and Internal Cuticle. *Second Chapter.*—Of the Nails and Hoofs. *Third Chapter.*—Of Hair and Feathers.

FIFTEENTH SECTION. Of the Bony System. *First Chapter.*—Of Bones in general. *Second Chapter.* Of Bones in particular.—A. Of the Bones of the Head.

B. Of the Bones of the Trunk. C. Of the Bones of the Upper Extremities. D. Of the Bones of the Lower Extremities.

SIXTEENTH SECTION. Of the Cartilaginous System.

SEVENTEENTH SECTION.—Of the Fibrous System in general, and of the Joints in particular.

EIGHTEENTH SECTION.—Of the Muscular System.

NINETEENTH SECTION.—Of the Vascular System. *First Chapter.*—Of the Pericardium. *Second Chapter.*—Of the Heart. *Third Chapter.*—Of the Arteries. *Fourth Chapter.*—Of the Veins. *Fifth Chapter.* Of the Lymphatic Vessels and Glands.

TWENTIETH SECTION.—Of the Nervous System. *First Chapter.*—Of the Brain. A. Of the Membranes of the Brain. B. Of the Brain itself. *Second Chapter.*—Of the Spinal Marrow. A. Of the Membranes of the Spinal Marrow. B. Of the Spinal Marrow itself. *Third Chapter.*—Of the Nerves."

As a specimen of the manner in which the subjects are handled, we subjoin the section on Alterations of Colour. Yet it is not a fair sample, for, independently of being selected at random, we are constrained for want of space to omit the referential notes, which equally evince the great labour of the author, and enhance the value of the work to the purchaser.

" *Of Vices relating to Colour.*

The IRREGULAR COLOURING of animal parts is very frequently conjoined with vices of texture, and may then consist, as in scirrhus, in the change into fat, in ossifications, &c. in diminution of colouring; or as in inflammations, caries, cancer, mortification, &c., in the deepening of colour, and frequently also in an entirely different colouring. But this abnormal colouring can also occur without any, or at least without any visible vice of texture, and then for the most part depends upon change in quantity or quality of the colouring fluids found in the organs, and especially of the blood.

First, as to what concerns diminished colouring; this is itself sometimes original; thus a particular part does not possess the proper degree of colour belonging to it, and remains pale as in the embryon, such is one kind of retarded formation; yet, however, deficiency of texture is usually connected with this state. To this belongs, also, the more common state of ALBINOISM, *leucæthiopia*, *leucopathia*, *albinoismus*, which occurs both in man and animals, in which the skin, the hair, and the eyes, are found unnaturally pale; this morbid state is sometimes hereditary in man, but more frequently in animals, and in the latter, then produces distinct varieties.

Unnatural paleness is more generally an acquired diseased state, a change of colour or bleaching, and is especially produced by a diminution of the blood generally, and of its *cruor* in particular, on which account it is the usual attendant of long continued disease, of cachexia, and especially of consumption and dropsy. To this place also we refer the more local change of colour, viz. in the skin of coloured persons, the change to grey and white of the hairs and feathers, the bleaching of dark eyes, &c.; the cause of this bleaching of the above-named parts is found in the diminution or total removal of the colouring matter. The frequent loss of colour in the mucous membrane of the mouth, and of the alimentary canal after poisoning with the concentrated acids and with tartar emetic, is also worthy of notice.

The abnormally increased colour of an organ, or the DEEPENING OF ITS COLOUR, depends in some cases, and especially in muscles originally pale, upon hypertrophy; but most commonly on the gorging of a part with blood, which has been augmented in it by congestion and stagnation, *stasis;* this is very often observed in the lungs, the liver, the spleen, and the mucous membrane of the alimentary canal. Sometimes it happens that the congested blood is itself unnaturally deep coloured, and thus doubly deepens the colour of the part in which it is collected; this is especially the case after suffocation, apoplexy, poisoning with narcotic substances, and hydrocyanic acid, adynamic fevers, and especially in the blue disease. A red or bluish colour is often observed in certain parts of the body, produced by congestion of blood in the most delicate vessels, for instance, DEATH SPOTS, *lividitas*, *nigror*, *sugillationes spuriæ*, the form, extent, and colour of which are very variable, according to circumstances; further, we notice similar red and dusky spots on the internal parts, arising from the same causes, as on the intestines, and more especially on their mucous coat; and the dusky colouring of those parts into which, if they be lowest, the blood sinks according to the laws of gravitation; such spots are produced, even sometime after death, on the putrefaction and thawing of frozen corpses.

From this kind of dusky spots we must distinguish other similar colourings which originate in the percolation of the darker juices after death; thus we sometimes observe in persons who have died of inflammation of the lungs accompanied with their adhesion to the pleura, large livid or violet-coloured spots on the chest; on the stomach there are seen dusky red spots, where it is in contact with the blood-gorged liver and spleen. The large venous trunks filled with blood often colour the neighbouring parts, and the gall-bladder very frequently tinges a part of the adjacent stomach and duodenum with its bile. Sometimes, also, the naturally pale surfaces immediately in contact with the blood become equally red, for instance, the inner surface of the heart, the great arterial trunks, the rectum in piles; a peculiar change in the blood seems to be the cause of this colouring.

A particular kind of deeper colouring is also produced by EXTRAVASATION, *ecchymosis*, *ecchymoma*, *effusio*, *suffusio*, *sugillatio*, either under the skin, or more deeply, but which differs from the above described similar spots, as it is produced by the actual effusion of blood from the vessels into the cellular membrane of the part. This usually accompanies a BRUISE, *contusio;* but it is not unfrequently consequent on severe strains of a part, on suckling in delicate skinned women, on great muscular exertions, on coughing, vomiting, and on many diseases, especially scurvy, petechial fevers, the morbus hæmorrhagicus of Werlhof, &c. These extravasations are at first blackish or blue, and distinctly circumscribed, but by degrees become more extended, as it were, fade, and by little and little assume a violet, greenish and yellow colour.

Further, the irregular colouring of animal bodies is often caused by the reception of various extraneous colouring matters into the body. This is observed almost generally in the lower animals, especially in the parasitic, when living on different kinds of food; also locally, as the consequence of various medicines and poisons; thus the bones of men, beasts and birds, are more or less reddened by food of a red colour. The taking of rhubarb often tinges light parts yellow; hydrocyanic acid renders many organs bluish or greenish; nitrate of silver, quack medicines, of which the composition is unknown, and other medicines, often render the skin blackish.

Finally, we oftentimes observe some peculiar colouring matter or pigment spontaneously produced in animal bodies, and colouring certain parts more or less completely; such is the case in jaundice, *icterus*, and in *melanosis*. In the former disease there is formed in the body of a yellow animal extractive or colouring matter, which has great similarity to the pigment of the bile, and tinges almost all the solid and fluid parts of the body more or less yellow and dusky, however, so that many of the systems assume a yellow colour more frequently than others. Diseases of the liver, asthenic fevers, as the plague, the American yellow fever, typhus fever, &c. are commonly attended with jaundice; the same also occurs in animals, but much less frequently. In *melanosis*, on the contrary, a deep brown or more frequently a blackish tinted pigment is morbidly produced, which occurs sometimes generally, sometimes to a limited extent; in the former, it is either mingled with the particular excretions, as the urine, the perspiration, the mucus from the lungs, in certain cases also, perhaps (in the matter evacuated) in *Melæna*, or the BLACK DISEASE of Hippocrates, and colours them blackish; or it may be deposited as a fluid or semi-coagulated mucus on the expanded surface of serous membranes, particularly the peritoneum and pleura. In the confined state the black pigment is found accumulated either in the otherwise healthy substance of an organ, especially of the skin, of the lungs, of the bronchial glands, &c. or it may accompany the various vices of texture, as mortification, particularly of the dry kind, false membranes, soft swellings, scirrhus, cancer, medullary sarcoma, and most commonly, at least in animals, tubercular swellings."

We again recommend this volume very cordially to the student, the surgeon, and physician. We have placed it in our own library as a standard work.

